# Psychometric properties of the 45-item supportive care needs survey—partners and caregivers - Dutch (SCNS-P&C45-D) in partners of patients with breast cancer

**DOI:** 10.1186/s41687-019-0092-7

**Published:** 2019-01-11

**Authors:** Mark J. A. Rietveld, Esmee J. Peters, Olga Husson, Floortje K. Ploos van Amstel, Y. Kamm, Sieta Sijtsema, Marieke Diepenbroek, Josca Heier, Celine Zoetbrood, Marijke Zielstra, Sylvie D. Lambert, Judith B. Prins, Petronella B. Ottevanger

**Affiliations:** 1 Department Of Medical Oncology, Radboud University Medical Center, Nijmegen, The Netherlands; 2 Department of Medical Psychology, Radboud University Medical Center, Nijmegen, The Netherlands; 3 Department of Clinical Studies, Institute of Cancer Research and Royal Marsden Hospital London, London, UK; 4 Department of Medical Oncology, Maasziekenhuis Pantein, Boxmeer, The Netherlands; 5 UMCUtrecht, Department of Medical Oncology, Utrecht, The Netherlands; 6 Department of Medical Oncology, Slingeland ziekenhuis, Doetinchem, The Netherlands; 7 Bernhoven, Department of Medical Oncology, Uden, The Netherlands; 8 Department of Medical Oncology, Gelre ziekenhuizen, Apeldoorn, The Netherlands; 9 VUmc, Department of Medical Oncology, Amsterdam, The Netherlands; 10 McGill University Medical Center, St. Mary’s Research Centre, Montreal, Canada; 11 Radboud university medical center, Geert Grooteplein Zuid 8, Postbus 9101, 6500 HB Nijmegen, The Netherlands

**Keywords:** Cancer, Oncology, Unmet needs, Spouse, Validation, Informal care

## Abstract

**Objective:**

To test the psychometric properties of the Dutch 45-item Supportive Care Needs Survey—Partners and Caregivers (SCNS-P&C45-D) among partners of women with breast cancer living in the Netherlands.

**Methods:**

In this cross-sectional validation study, partners of patients with breast cancer were invited to complete a survey on the patient’s cancer and the caregiver’s level of unmet needs (SCNS-P&C45-D), psychological distress (HADS) and burden (EDIZ).

**Results:**

43% of the invited informal caregivers responded (*n* = 302). Flooring effects were identified for three items of the SCNS-P&C45-D,which were then deleted from further analysis. The original factor structure and loading pattern of the SCNS-P&C45-D was not replicated. Internal consistency of the SCNS-P&C45-D and all subscales’ (emotional and relational needs, health care and illness related needs, practical needs, work and social needs) Cronbach’s alpha coefficients exceeded 0.80, the entire measure’s Cronbach’s alpha is 0.98. Most SCNS-P&C45-D subscales showed moderate correlations with distress and burden from informal care which was in line with expectations based on validity. The domain ‘Work and Social needs’ showed a high correlation with burden from informal care. Participants reported significantly more or higher unmet needs if they were younger (25.5% vs. 20.3% in older patients, *p* = 0.004), if diagnosis was less than 1 year ago in one subscale (Health Care and Illness related needs; 19.5% and 18%, *p* = 0.029, and the total SCNS-P&C45-D; 23.2% vs. 22.4%, *p* = 0.018).

**Conclusions:**

The SCNS-P&C45-D is able to identify those partners of patients with breast cancer in need and those who are not.

**Electronic supplementary material:**

The online version of this article (10.1186/s41687-019-0092-7) contains supplementary material, which is available to authorized users.

## Introduction

Patients with breast cancer (BC) often rely on their partners as the primary source of support [1–3]. Common additional responsibilities for partners include assisting patients with daily activities, providing emotional support, helping them with managing tasks related to the illness or treatment and advocating for patients with health care team [2, 4]. These caring responsibilities are often faced without applicable knowledge or additional support [4, 5]. During BC treatment, partners’ distress levels are higher than normal population values with clinically significant distress levels ranging from 10 to 60% [2, 6–8]. Partners’ distress levels are also higher than what patients have been found to report [1, 2, 5, 7, 9]. In general, partners’ key concerns are related to supporting the cancer patient which can result in partners ignoring their own worries, needs and having a lower quality of life (QoL) [1–3, 9, 10]. The impact of BC on partners could impair patients’ adjustment [1, 4, 5, 7, 10]. Partners who report more needs are known to provide less support to patients, and partners’ unsupportive reactions are associated with social and emotional problems for both the patient and the partner [3–5, 10, 11]. However, positive support by partners can help patients to adjust and improve their QoL [3–5, 7, 10, 11].

An important first step towards designing effective health care services for partners of patients with BC is an assessment [12, 13] of the physical, informational, emotional, practical, social and spiritual supportive care needs of the partner [14, 15]. Unmet supportive care needs are typically defined as the discrepancy between the required service or support that is necessary to do well and the actual received service or support [14]. Assessing unmet supportive care needs can reveal areas where patients or partners require help, and could therefore improve care [16]. Studies using different measures report that 19–82% of the partners report at least one unmet need [1, 12, 17]. It is suggested that needs of patients with cancer and their partners are partly similar but may also differ from each other [6]. Reported unmet needs of partners include health care services, emotional and psychological aspects, relationships, impact on daily activities (including socio-economic issues or work), life expectations and spirituality [1, 5, 7, 10, 17–19].

Currently, no psychometrically sound tool to measure unmet supportive care needs in partners of patients with cancer is available in the Netherlands. To contribute to the literature of unmet needs in partners of patients with cancer and to capture possible cultural differences, it is important to have a Dutch validated questionnaire. The Supportive Care Needs Survey— Partners and Caregivers (SCNS-P&C) has been used in several studies [9, 13]. It has an adequate internal consistency (Cronbach’s α = 0.88–0.94) and provides a comprehensive assessment of the multi-dimensional supportive care needs of partners of cancer patients, across the illness trajectory [19]. We expect psychometric properties of the Dutch questionnaire to be comparable to the original Australian version. This article reports on the psychometric testing of the Dutch SCNS-P&C45 (SCNS-P&C45-D).

## Methods

### Procedure

**Fig. 1:**
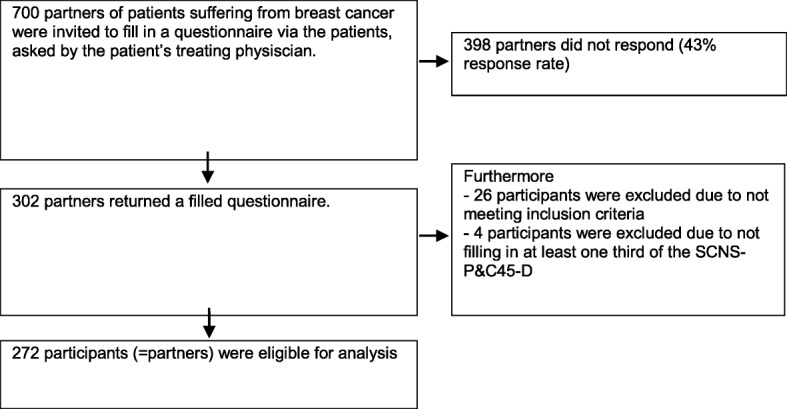
Flowchart of the data collection process

Partners of patients with BC (hereafter named participants) were invited to participate in this cross-sectional psychometric study,. The method of inviting participants varied based on the preferences of the hospital. In five hospitals, participants were invited either directly via a research nurse or indirectly via the patient who was asked by a nurse to hand over the questionnaire to their partner. All participants were asked to return the questionnaires to the researcher (EP) in a Reply Paid envelope (Fig. 1).

### Participants

To be eligible, participants had to be 18 years or older and have an intimate relationship with a patient diagnosed with BC who was receiving curative treatment or had received primary treatment ≤5 years ago. All participants were native Dutch speakers who could adequately read, speak and understand Dutch. We specified no criteria regarding the patient’s and partner’s gender since breast cancer is very rare in males.

### Data collection

#### Supportive care needs survey

The Supportive Care Needs Survey—Partners and Caregivers (SCNS-P&C45) consists of 45 items that measure caregivers’ unmet needs [20]. Participants indicate their level of need for help in the last month on a 5-point scale. The response scale distinguishes between no needs (1 not applicable, 2 fulfilled needs) and unmet needs (3 low, 4 moderate, 5 high). The original factor analysis revealed four domains of needs (including 39 items): 1) health care service needs; 2) psychological and emotional needs; 3) work and social needs and 4) information needs. The English-Dutch forward-backward translation of the SCNS-P&C45 involved four people, two native Dutch-speaking and two native English-speaking, all fluent in both languages. Initial translated items were reviewed for face and content validity by experts in psycho-oncology (*n* = 8), partners of patients with BC (*n* = 7) and members of the general public as a control group (*n* = 9). Evidence for the face validity and content validity was supported by subjective feedback on an evaluation sheet. Based on these data some changes to the original questionnaire were made: 1) we added ‘in the last month’ in the explanation of every response category; 2) we used ‘patient with cancer’ instead of ‘person with cancer’; 3) we deleted the examples; 4) we changed item 8 (accessing local health care services when needed) into ‘involving the General Practitioner in the care process’ and 5) we changed item 27 (communicating with the family) into ‘communicating with (grand)children’. In addition, some minor textual suggestions were integrated to improve the understanding of the Dutch version of the survey (SCNS-P&C45-D).

### Other measures for discriminant and convergent validity

Partners’ demographics and questions related to the patients’ illness such as time since diagnosis and type of treatments were reported. The Hospital Anxiety and Depression scale (HADS) was used to examine psychological distress and to determine convergent validity [21]. Each of the 14-items was rated on a scale from zero (not at all) to three (very much). Higher scores imply more psychological distress. The total score was used to describe psychological distress with a cut-off point of 11 [21]. The HADS revealed Cronbach’s alpha’s from 0.71 to 0.90 for the total scale and both subscales [21]. The EDIZ (Self-Perceived burden from Informal Care) is a Dutch instrument to measure the self-perceived burden from informal care with 9-items on a 5-point Likert scale. This instrument was validated for partners of patients with dementia [22] and also used for partners of cancer patients in palliative care [23]. Higher scores imply higher burden due to informal care. The EDIZ revealed Cronbach’s alpha of 0.79 [23]. The EDIZ was used to determine convergent validity too.

### Ethical considerations

Permission to conduct the study was obtained from the regional ethics review board (Radboud university medical center, no. 2013–350). The committee concluded that no detailed ethical approval was necessary. Returning the questionnaire was considered as an implicit informed consent. All procedures performed in studies involving human participants were in accordance with the ethical standards of the institutional and/or national research committee and with the 1964 Helsinki declaration and its later amendments or comparable ethical standards.

### Data analysis

Psychometric analyses of the SCNS-P&C45-D were performed using SPSS 22 (SPSS Inc., Chicago, IL, USA). When a participant left more than 33.3% of the SCNS-P&C45 questions blank, the participant was excluded from further analysis due to lacking data. This was done to maintain the reliability of remaining data. Remaining missing data were omitted per item, leading to a fluctuating number of participants answering a certain item. Descriptive statistics were calculated to identify the mean distress level, caregiving burden and mean number of unmet needs. Unmet needs were defined as moderate (response 4) or high (response 5) needs. For data analysis of the EDIZ, the 5-point scale was recoded to a dichotomous score. Score 1–2 (no) were recoded in score 0, score 3–5 (yes) were recoded in score 1. Total scores 0–3 indicate little self-perceived burden, scores 4–6 moderate self-perceived burden and scores 7–9 many self-perceived burden [22]. For the descriptive statistics of the SCNS-P&C45-D we focused on the mean number of moderate/high unmet needs (response category 4 and 5), consistent with previous research [12]. Following the example of Girgis et al. (2011) the responses 1 and 2 were re-coded as 1 ‘No need’ and the other response categories were re-scored (2–4) to ensure a linear response format for the factory analysis [18]. As in the study of Girgis et al. (2011), flooring effects were identified with descriptive statistics, if more than 90% of the participants answered ‘no need’, that item was deleted from further analysis [18]. To identify underlying need domains of the SCNS-P&C45-D, exploratory factor analysis was used. To discover the suitability of the data, the correlation matrix was inspected for extreme high (*r* > .90) or no significant correlations [24]. The Kaiser–Meyer–Olkin (KMO) measure of sampling adequacy (>.6) and Bartlett test of Sphericity (*p* < 0.05) were calculated to test whether the sample size was appropriate [24]. Principal component analysis (PCA) was performed using oblique rotation, because of expected inter-correlations [24]. Factors were identified using Kaiser Criterion (based on Cattell’s scree plot and eigenvalues of 1.0 or greater) [18]. Items were included in a factor if: 1) the minimum loading was > 0.30; 2) the items scored highest on this certain factor; and 3) the factor contained at least three items [25]. Internal reliability of each subscale was calculated using Cronbach α, correlations between 0.70–0.95 were preferred [26]. Missing values were managed using listwise deletion in the factor analysis.

Construct validity was measured with convergent and discriminant validity [27]. Validity refers to whether an instrument actually measures what it is supposed to measure [28]. Validity was measured by calculating Pearson correlation coefficients for the SCNS-P&C45-D with distress (HADS) and burden from care (EDIZ). Many studies suggested that more needs were reported by participants with higher levels of distress or more burden from informal care [12, 13]. In concordance with these results, it was hypothesized that the number of total needs (moderate or high needs) was correlated with the total score on the HADS and with the total score on the EDIZ (Pearson’s *r* > .30: moderate; Pearson’s *r* > .60: large) [29]. It was expected that HADS and EDIZ scales that are conceptually related correlate moderately to highly with one another (*r* ≥ 0.40). Conversely, scales with a less conceptual relation are expected to show weak correlations (*r* < 0.40) [27]. We tested known-group validity, as a form of discriminant validity, in different groups of which higher needs were expected based on the literature. Although not consistent, studies have shown that those having more needs are often younger [12, 18]. Our hypothesis was that participants younger than sixty would report more often at least one unmet moderate or high need across domains. The cut-off point of 60 years of age was chosen based on the literature that describes significant relationships between age and unmet needs of informal caregivers [18]. Although unmet needs are not uncommon in (long-term) survivors and their informal caregivers [1, 15, 17, 18], a cohort study [30] and a longitudinal study [12] showed that unmet needs decrease in the course of time since diagnosis. Our hypothesis was that partners of patients who were diagnosed with BC less than one year ago, more often had at least one unmet moderate or high need than those diagnosed more than one year ago.

## Results

### Participants

Three hundred two participants answered the survey (700 invited, response rate = 43%), 26 participants were excluded because of predetermined illness exclusion criteria (e.g. recurrence; metastases; > 5 year after diagnosis). Four surveys had more than 33.3% items of the SCNS-P&C45-D missing and were therefore excluded from further analysis, resulting in 272 participants eligible for analysis.

The demographic characteristics of the participants are reported in Table 1.

**Table 1:** Participants’ demographics and patients medical characteristics (*n* = 272)

Participants’ demographics		No. (%)
Nationality (Dutch)		271 (99.6%)
Gender	Man	265 (97.4%)
	Women	7 (2.6%)
Gender patient	Women	270 (99.3%)
	Man	2 (0.7%)
Age, mean (SD), years (*n* = 271)		61.4 (10,6)
Married and/or cohabited		265 (97.4%)
Duration of the relationship	1–5 years	10 (3.7%)
	6–10 years	12 (4.4%)
	11–20 years	34 (12.5%)
	> 20 years	216 (79.4%)
Children	Yes, living independently	155 (57%)
	Yes, living at home	77 (28.3)
	No	37 (13.6)
	No, but a desire to have children	3 (1.1)
Education^a^, mean (SD) (n = 271)		4.99 (1.4)
Occupation^b^ (*n* = 271)	Paid work	141 (52%)
	Retirement	114 (42.1%)
	Disablement insurance act or sick leave	12 (4.4%)
	Other	21 (7.7%) 7,7
Type of hospital	General hospitals	142 (52,2%)
	University hospitals	130 (47,8%)
Medical characteristics of the patients		No. (%)
Time since diagnosis (*n* = 272)	In the last month	2 (0.7%), 7
	1–6 months	50 (18.4%)
	6–12 months	65 (23.9%)
	1–2 year	83 (30.5%)
	2–5 year	72 (26.5%)
Received treatment (*n* = 270)	Radiotherapy (RT)	171 (63.3%) 63,3
	Chemotherapy (CT)	131 (48.5%) 48,5
	Immunotherapy (IT)	12 (4.4%) 4,4
	Hormonal therapy (HT)	3 (1.1%) 1,1
	No addition therapy finished	44 (16.3%) 16,3
Current treatment (*n* = 270)	(RT or CT or IT or HT)	159 (58.9%) 58,9
	(RT or CT or IT)	46 (17%) 17,0
	(RT or CT)	36 (13.3%) 13,3

^a^ According to the Dutch standardized scoring system (range 1–7) in which 1 is no education and 7 is university

^b^ Multiple answers possible, answers do not app up to 100%

### Factor analysis

All items on the SCNS-P&C45-D were significantly correlated, correlations ranged between *r* = .13 and *r* = .85. The KMO statistic (.94) was above the recommended value of .6, Bartlett’s test was significant (χ^2^(861) = 11,588.67, *p* < 0.001). Flooring effects were identified for item 18 (Accessing information about possible fertility problems of the patient with cancer) (96.7% no need), 19 (Looking after the patient with cancer) (93.4% no need) and 43 (Exploring your spiritual beliefs) (92.6% no need). These items were deleted from further factor analyses. The original factor structure and loading pattern of the SCNS-P&C45 was not replicated. The PCA with oblimin rotation showed five factors meeting Kaiser’s eigenvalue criterion (above 1), explaining 72% of the total variance (Additional file 1: Table S1).

One factor included one item (item 4: information concerning alternative therapies) which was therefore transferred to the factor with the second highest loading (factor 2). The four remaining factors explained 69.4% of the variance.

Factor 1 explained 55% of variance and includes 14 items regarding “Emotional and relational needs”.

The 16 items concerning “Health Care and Illness related needs” included in factor 2 accounted for 7.5% of the total variance. Due to cross loading and a better conceptual fitting, item 15 ‘look after own health’ and item 1 ‘caregiver information needs’ were replaced into factor 1.

Factor 3 included 4 items regarding “Practical needs” explaining 3.6% of variance.

Factor 4 relates to “Work and Social needs” which accounted for 3.3% of variance and included 7 items.

### Reliability

Internal consistency of the SCNS-P&C45-D and all subscales proved high, with Cronbach’s alpha coefficients ranging from 0.82 (*Practical needs*) to 0.97 (*Health Care and Illness related needs*). Cronbach’s alpha of the SCNS-P&C45-D is 0.98 (Table 2).

**Table 2:** Intercorrelations between SCNS-P&C45-D subscales and total SCNS-P&C45-D score, no. of items, mean (SD), Cronbach’s alpha and percentage of variance

Correlations					Subscale details				
	Emotional and relational needs	Health Care and Illness related needs	Practical needs	Work and Social needs	No. of Items	N	Mean (SD)	Range of Scores	Cronbach’s alpha
Psychosocial and Emotion needs					16	272	22.2 (10.3)	16–58	0.96
Health Care and Illness related needs	.772				15	267	22.1 (11.4)	15–60	0.97
Practical needs	.584	.591^**^			4	272	5.2 (2.4)	4–16	0.82
Work and Social needs	.747	.670^**^	.537		7	272	8.8 (4)	7–28	0.92
Total SCNS-P&C45-D	.925	.919	.661	.737	45	272	61.4 (26.08)	45–180	0.98

Spearman’s correlation coefficient was calculated; All correlation were significant (*p* < .001)

Mean using factor response format 1–4: 1 = “No need – not applicable and No need - satisfied” to 4 = “High need for help”

### Convergent validity

Moderate to high correlations between the total SCNS-P&C45-D and the HADS-T and EDIZ total score were found (*r* = .478 and *r* = .521, respectively; *p* < 0.001). Most SCNS-P&C45-D subscales showed moderate correlations with distress and burden from informal care, ranging from *r* = .32 (Practical needs) to *r* = .48 (Emotional and relational needs). The domain ‘Work and Social needs’ showed a high correlation (.523) with burden from informal care (EDIZ) (Table 3).

**Table 3:** Correlations between the SCNS-P&C45-D domains and distress and Self-Perceived Burden from Informal Care (*n* = 272)

	Emotional and relational needs	Health Care and Illness related needs	Practical needs	Work and Social needs	Total SCNS-P&C45-D
Distress	.493	.375	.320	.356	.478
Self-Perceived Burden from Informal Care	.490	.462	.340	.523	.521

Spearman’ correlation coefficient was calculated; All correlation were significant (p < .001)

### Discriminant validity

All domains and the total SCNS-P&C45-D showed a significantly greater proportion of younger participants (< 60 years) (mean age 52, median 53 and range 25–60 years) with at least one unmet moderate or high need in comparison to older participants (≥60 years) (mean age 69, median 68 and range 60–93 years) (SCNS-P&C45-D 25.5% and 20.3%, respectively *p* = 0.004).. Partners of patients diagnosed less than 1 year ago, reported slightly more often, but significantly one unmet moderate or high need in the Health Care and Illness related needs subscale in comparison with partners of patients diagnosed more than 1 year ago, significantly; 19.5% and 18%, respectively, *p* = 0.029, and the total SCNS-P&C45-D; respectively 23.2% and 22.4%, *p* = 0.018). In the domains of Emotion and Relational needs, Work and Social needs and Practical needs, no significant differences were found between participants whose partners were diagnosed less than 1 year ago and those of whose partners were diagnosed more than 1 year ago (Additional file 2: Table S2).

## Discussion

In this cross-sectional study, we tested the psychometric properties of the SCNS-P&C45-D in a sample of predominantly male partners of BC patients in the Netherlands. The psychometric properties of the SCNS-P&C45-D were acceptable. The face validity of the survey was supported by experts in psycho-oncology, partners of patients with BC and members of the general public. Due to the cross-sectional nature of the study, we were not able to measure test-retest reliability. Internal consistency of the SCNS-P&C45-D and all subscales was within the acceptable range [18]. We found four meaningful factors for the SCNS-P&C45-D, namely “Emotional and relational needs”, “Health care and illness related needs”, “Practical needs” and “Work and social needs”. These factors echo the domains found in the caregiver literature [6, 17–19]. The four factors explain 69.4% of the variance. The original factor structure and loading pattern of the SCNS-P&C45 were not replicated [18]. We believe this might be related to cultural differences and/or difference in responses group (only men with a partner with BC vs. men and women across cancer types). A remarkable difference between the Australian and the Dutch version of the survey is the difference in excluded items for factor analyses based on flooring effects (> 90% no need). In the original factor analysis item 15 “looking after own health”, item 18 “receiving information about fertility problems in patient”, item 19 “take care of the patient with cancer”, item 24 “insurance for patient” and item 25 “access to legal services” were excluded from further analysis. In the Dutch survey, also item 18 “receiving information about fertility problems in patient” and item 19 “take care of the patient with cancer” were excluded. In addition, item 43 “discover own spiritual beliefs” was excluded. We can think of several explanations for these differences when comparing the Australian and Dutch version. First, this might be related to cultural differences and/or difference in responses group as mentioned earlier. Furthermore, this might be due to age differences. We included patients aged 25–93, where the Australian authors included patients aged 16–85, as younger patients report more and other needs [15]. Furthermore, the Australian version included different types of cancer while the Dutch version included only partners of BC patients. Moreover, differences may occur due to translation and interpretation of questions and answers [31, 32]. Also, self-reported needs can be an overestimation or underestimation of health because patients’ understanding of health status may not necessarily correspondent with the appraisal of the health care professional [32, 33]. Health literacy could also play a role; low literacy levels may correlate with the inability to process the essential information in the questionnaires [33, 34]. Furthermore, differences might be due to sample size, where the Australian version included 547 participants, the Dutch variant included 302 participants. In general, we believe that the high number of participants answering an item with ‘no need’ should not directly be seen as a flooring effect. One of the clinical applications of the SCNS-P&C45-D is to identify partners who are in need of support and those who are not. In this study, 54.4% of the participants did not report any moderate or high needs. Because there was no ceiling effect found, the survey is able to identify those in need and those who are not. Further outstanding difference in the Dutch and Australian factors is the not existing “Information needs” factor in the Dutch survey. In the Dutch version, information needs are more scattered over the four factors. In the Dutch questionnaire however, the appeared to be a factor related to the more practical needs of partners instead. Lastly, the Dutch version included only partners of patients, whereas the Australian version of the survey included other informal caregivers too [18].

Internal consistency of the SCNS-P&C45-D and all subscales proved good, though somewhat high, with Cronbach’s alpha coefficients ranging from 0.82 (*Practical needs*) to 0.98 (*total* SCNS-P&C45-D). In the original survey, a high internal consistency was found as well. Often, coefficients above 0.95 indicate that several items measure the same construct and may be redundant. This suggests that future research could look into a shorter version of the survey. This was also the case with the patient version of this questionnaire [35].

### Clinical implications

The hypotheses concerning the convergent validity of the SCNS-P&C45-D were confirmed and partially confirmed concerning discriminant validity. The total SCNS-P&C45-D score was moderate/high positively correlated with the level of distress and burden from informal care. These relations have been found in other studies as well (for example in brain-, esophageal-, lung-, prostate-, testicular-, ovarian-, bowel-, and head and neck-cancer) [9, 36–38]. Experiencing any need or even just one can be quite distressing. All domains of the questionnaire had a moderately positive correlation with the distress and burden from informal care. All domains and the total SCNS-P&C45-D showed a significantly greater proportion of younger participants (< 60 years old) with at least one unmet moderate or high need in comparison to older participants. Other studies support this finding [1, 8, 12, 15]. Participants whose partner was diagnosed less than 1 year ago, often showed one or more unmet moderate or high need in the subscale of Health Care and Illness related needs, and overall at the SCNS-P&C45-D. Health care professionals should be aware of present but latent unmet needs of the patients and the informal caregiver, especially for younger patients and informal caregivers that might have more needs than older informal caregivers. During consultation not only the patient, but the informal caregiver should regularly be asked if the information was understandable, helpful and whether personal needs have been met in order to unravel persisting unmet needs.

### Study limitations

This study has a number of strengths: the sample size, the distribution of age and the time since diagnosis. There is a good diversity between the type of hospitals and the regional spread of these hospitals in the Netherlands. In addition, we had a sufficient response rate. However, our study focused only on the intimate partner of the cancer patient and not on informal care givers in general. In addition, our sample was limited to (predominantly) male partners of breast cancer patients. Further study should validate the survey in partners of patients with other cancer types and stages. Future research on female patients with female informal caregivers should be performed to explore if the current factor structure is also valid for female partners of women suffering from BC. To expand on the psychometric properties of the SCNS-P&C45-D, further studies should investigate other indicators such as reproducibility of responsiveness measured by test-retest reliability and minimal clinical important difference [9, 27]. Since we used paper and pencil questionnaires we had some missing data. There is the also the possibility of selection bias as we do not know why the participating partners filled in the questionnaires and others did not. In addition, with the questionnaires sent by mail we had no control of whether the questionnaires were actually received by the partner of the patient. Lastly, in our study recall bias may have occurred since a considerable proportion of the population has been in follow-up longer than two years. Further research should investigate unmet needs of informal caregivers of patients that are in the early follow-up phase.

## Conclusion

In conclusion, we believe this validation study showed that the SCNS-P&C45-D is capable of measuring the unmet needs of partners of breast cancer patients during the course of this disease.

## Additional files

Additional file 1: **Table S1.** Scale and item characteristics of the SCNS-P&C45-D. (DOCX 23 kb)

Additional file 2: **Table S2.** Percentage participants with at least one unmet moderate/high need by age and time since diagnosis (*n* = 272). (DOCX 14 kb)

## Abbreviations

BC: Breast cancer
EDIZ: Self-Perceived burden from Informal Care
HADS: Hospital Anxiety and Depression scale
KMO: Kaiser–Meyer–Olkin
PCA: Principal component analysis
QoL: Quality of life
SCNS-P&C: Supportive Care Needs Survey— Partners and Caregivers
SD: Standard deviation
