# Supplementary material for: Psychometric properties of the 45-item supportive care needs survey—partners and caregivers - Dutch (SCNS-P&C45-D) in partners of patients with breast cancer
Source: J Patient Rep Outcomes. 2019 Jan 11;3:1. doi: 10.1186/s41687-019-0092-7 (PMC6329686; doi:10.1186/s41687-019-0092-7)
Supplement: Supplementary file 1 — Table S1. Scale and item characteristics of the SCNS-P&C45-D. (DOCX 23 kb) [file 41687_2019_92_MOESM1_ESM.docx]

|  | **Table S1: Scale and item characteristics of the SCNS-P&C45-D** | | | | | | | | | | | |
| --- | --- | --- | --- | --- | --- | --- | --- | --- | --- | --- | --- | --- |
| *How much help did you need in the last month with* | | **N** | **Mean** | **SD** | **Missing N (%)** | **No Need (%)** | **Moder te-**  **high (%)** | **Factor loadings** | | | | |
|  |  |  |  |  |  |  |  | **Factor 1** | **Factor 2** | **Factor 3** | **Factor 4** | **Factor 5** |
| 1_Accessing information that is easy to understand | | 265 | 1.27 | 0.71 | 7 (3) | 84.9% | 7.9% | .581 | **-.681** | .493 | **.678** | .483 |
| 2_Accessing information on the prognosis of the patient with cancer | | 265 | 1.46 | 0.95 | 7 (3) | 77.7% | 15.5% | .421 | **-.822** | .437 | .449 | .457 |
| 3_Accessing information on supporting services for caregivers or partners | | 266 | 1.27 | 0.69 | 6 (2) | 84.2% | 7.9% | .656 | **-.683** | .458 | .679 | .488 |
| 4_Accessing information on alternative therapies | | 265 | 1.18 | 0.52 | 7 (3) | 87.2% | 5.3% | .410 | **-.451** | .319 | .423 | **.546** |
| 5_Accessing information on what the physical needs of the patiënt with cancer are likely to be | | 267 | 1.42 | 0.86 | 5 (2) | 77.2% | 13.1% | .496 | **-.726** | .450 | .498 | .630 |
| 6_Accessing information on the advantages and side effects of treatments | | 265 | 1.54 | 0.98 | 7 (3) | 72.5% | 18.5% | .480 | **-.853** | .463 | .483 | .464 |
| 7_Getting the best medical care for the patient with cancer | | 266 | 1.59 | 1.08 | 6 (2) | 74.4% | 19.9% | .462 | **-.893** | .491 | .486 |  |
| 8_Involving the GP in the care process | | 266 | 1.45 | 0.91 | 6 (2) | 76.7% | 14.3% | .483 | **-.790** | .448 | .580 |  |
| 9_Involvement in the care of the patient with cancer, together with the medical team | | 265 | 1.47 | 0.93 | 7 (3) | 76.6% | 16.6% | .403 | **-.906** | .424 | .524 |  |
| 10_Having opportunities to discuss your concernswith the doctors | | 267 | 1.50 | 0.96 | 5 (2) | 74.9% | 17.2% | .456 | **-.902** | .452 | .512 |  |
| 11_Feeling confident that all the doctors consult with each other in order to coordinate care of the patient with cancer | | 265 | 1.55 | 1.05 | 7 (3) | 75.5% | 18.9% | .438 | **-.923** | .465 | .519 |  |
| 12_Getting certainty about the fact that there was always one case manager who coordinated the services to the patient with cancer | | 267 | 1.51 | 1.01 | 5 (2) | 76.8% | 16.9% | .513 | **-.892** | .513 | .572 |  |
| 13_Ensuring that any complaints about the care of the patient with cancer were dealth with appropriately | | 266 | 1.61 | 1.08 | 6 (2) | 72.2% | 19.5% | .524 | **-.893** | .407 | .515 |  |
| 14_Reducing stress in the life of the patient with cancer | | 266 | 1.74 | 1.11 | 6 (2) | 64.7% | 24.8% | .658 | **-.839** | .471 | .468 |  |
| 15_Looking after your own health | | 267 | 1.49 | 0.92 | 5 (2) | 74.2% | 16.1% | **.656** | **-.725** | .396 | .531 |  |
| 16_Coordinating of adequate pain control for the patient with cancer | | 272 | 1.45 | 0.94 | 0 (0) | 78.7% | 15.8% | .638 | **-.735** | .688 | .584 |  |
| 17_To address fears of the physical and mental detoriation of the patient with cancer | | 272 | 1.40 | 0.83 | 0 (0) | 77.6% | 13.2% | **.688** | -.658 | .612 | .564 |  |
| 18_Accessing information about possible fertility problems of the patient with cancer | | 271 | 1.05 | 0.29 | 1 (0) | 96.7% | 1.1% |  |  |  |  |  |
| 19_Looking after the patient with cancer | | 272 | 1.12 | 0.50 | 0 (0) | 93.4% | 4.0% |  |  |  |  |  |
| 20_Finding better accessible parking space at the hospital | | 271 | 1.30 | 0.79 | 1 (0) | 85.6% | 10.0% | .391 | -.339 | **.652** | .408 |  |
| 21_Adjustment to changes in the working life or usual activities of the patient with cancer | | 272 | 1.26 | 0.68 | 0 (0) | 84.9% | 8.1% | .489 | -.520 | .412 | **.844** |  |
| 22_The influence caring for the patient with cancer on your working life or usual activities | | 272 | 1.26 | 0.72 | 0 (0) | 85.3% | 7.7% | .402 | -.501 | .442 | **.851** |  |
| 23_Finding out about financial support and governmental benefits | | 271 | 1.43 | 0.91 | 1 (0) | 79.0% | 14.4% | .437 | -.545 | **.881** | .456 |  |
| 24_Getting life and/or travel Insurance fpr the patient with cancer | | 272 | 1.21 | 0.68 | 0 (0) | 89.3% | 6.6% | .388 | -.429 | **.801** | .330 |  |
| 25_Accessing legal services | | 271 | 1.21 | 0.62 | 1 (0) | 88.2% | 7.4% | .495 | -.490 | **.797** | .470 |  |
| 26_Communicating with the patient with cancer | | 271 | 1.29 | 0.75 | 1 (0) | 85.2% | 10.0% | .618 | -.634 | .389 | **.688** |  |
| 27_Communicating with (grand)children | | 271 | 1.29 | 0.76 | 1 (0) | 85.2% | 10.3% | .683 | -.650 | .399 | **.763** |  |
| 28_Getting more support from your family | | 271 | 1.22 | 0.63 | 1 (0) | 87.% | 6.6% | .682 | -.534 | .441 | **.721** |  |
| 29_Talking to others who have looked after a patient with cancer | | 271 | 1.23 | 0.60 | 1 (0) | 84.5% | 6.3% | .649 | -.496 | .404 | **.831** |  |
| 30_Dealing with the ‘issue of cancer’ in social situations or at work | | 272 | 1.24 | 0.65 | 0 (0) | 85.7% | 7.4% | .612 | -.470 | .441 | **.800** |  |
| 31_Dealing with concerns whether the cancer will come back | | 271 | 1.55 | 0.91 | 1 (0) | 67.5% | 16.6% | **.690** | -.637 | .571 | .515 |  |
| 32_The influence cancer has had on your relationship with the patient with cancer | | 272 | 1.42 | 0.85 | 0 (0) | 76.1% | 13.2% | **.853** | -.577 | .339 | .607 |  |
| 33_Understanding the experiences of the patient with cancer | | 272 | 1.49 | 0.91 | 0 (0) | 73.9% | 16.5% | **.825** | -.617 | .315 | .668 |  |
| 34_Finfing the balance between the needs of the patient with cancer and your own needs | | 272 | 1.50 | 0.91 | 0 (0) | 72.8% | 18.0% | **.881** | -.542 | .419 | .606 |  |
| 35_Adjustment to changes in the body of the patient with cancer | | 272 | 1.40 | 0.83 | 0 (0) | 78.3% | 14.3% | **.859** | -.516 | .487 | .521 |  |
| 36_Addressing problems in your sex life | | 271 | 1.41 | 0.83 | 1 (0) | 76.8% | 12.9% | **.687** | -.310 | .359 | .348 | .377 |
| 37_Getting emotional support for yourself | | 271 | 1.34 | 0.75 | 1 (0) | 79.3% | 9.6% | **.841** | -.448 | .384 | .577 |  |
| 38_Getting emotional support for the people you love | | 272 | 1.32 | 0.76 | 0 (0) | 82.0% | 11.0% | **.878** | -.545 | .449 | .584 |  |
| 39_Dealing with your emotions about death and dying | | 272 | 1.37 | 0.77 | 0 (0) | 77.9% | 11.4% | **.838** | -.478 | .416 | .465 |  |
| 40_Dealing with others who don’t recognize the effects on your life of looking after the patient with cancer | | 272 | 1.36 | 0.77 | 0 (0) | 77.9% | 11.0% | **.751** | -.407 | .524 | .493 |  |
| 41_Dealing with your emotions when the recovery of the person with cancer has not happened as you had expected | | 271 | 1.26 | 0.67 | 1 (0) | 84.9% | 9.2% | **.693** | -.519 | .480 | .476 |  |
| 42_Taking decisions about your life in the midst of uncertainty | | 271 | 1.28 | 0.72 | 1 (0) | 84.5% | 9.6% | **.761** | -.412 | .546 | .385 |  |
| 43_Exploring your spiritual beliefs | | 272 | 1.11 | 0.41 | 0 (0) | 92.6% | 2.9% |  |  |  |  |  |
| 44_Being able to give meaning to the illness of the patient with cancer | | 272 | 1.29 | 0.70 | 0 (0) | 82.0% | 8.1% | **.835** | -.541 | .476 | .636 |  |
| 45_The opinion to influence decisions in the treatment of the patient with cancer | | 270 | 1.34 | 0.77 | 2 (1) | 80.7% | 11.9% | .635 | **-.675** | .528 | .415 |  |

Extraction Method: Principal Component Analysis. Rotation Method: Oblimin with Kaiser Normalization. Structure matrix. Showing factor loadings > 0.3. Mean using factor response format 1-4: 1= “no need – not applicable and no need - satisfied” to 4 = “high need for help”
