# Supplementary material for: Psychometric properties of the 45-item supportive care needs survey—partners and caregivers - Dutch (SCNS-P&C45-D) in partners of patients with breast cancer
Source: J Patient Rep Outcomes. 2019 Jan 11;3:1. doi: 10.1186/s41687-019-0092-7 (PMC6329686; doi:10.1186/s41687-019-0092-7)
Supplement: Supplementary file 2 — Table S2. Percentage participants with at least one unmet moderate/high need by age and time since diagnosis (n = 272). (DOCX 14 kb) [file 41687_2019_92_MOESM2_ESM.docx]

| **Table S2: Percentage participants with at least one unmet moderate/high need by age and time since diagnosis (n=272)** | | | | | | |
| --- | --- | --- | --- | --- | --- | --- |
|  | | ***Emotional and relational needs*** | ***Health Care and Illness related needs*** | ***Practical needs*** | ***Work and Social needs*** | **Total Needs /SCNS-P&C45-D** |
| **Age** | <60 years old | 19.6 | 19.9 | 11.1 | 10.3 | 20.3 |
|  | ≥60 years old | 14.4 | 17.7 | 8.1 | 5.5 | 25.5 |
|  | p-value | .002 | .028 | .035 | .003 | .001 |
| **Time**  **since diagnose** | ≤ 1 year | 16.2 | 19.5 | 8.1 | 8.8 | 23.2 |
|  | 1-5 year | 17.6 | 18 | 11 | 7 | 22.4 |
|  | p-value | .252 | .029 | .909 | .065 | .018 |

Chi-square test
